# X-ray structural analyses of azide-bound cytochrome *c* oxidases reveal that the H-pathway is critically important for the proton-pumping activity

**DOI:** 10.1074/jbc.RA118.003123

**Published:** 2018-08-03

**Authors:** Atsuhiro Shimada, Keita Hatano, Hitomi Tadehara, Naomine Yano, Kyoko Shinzawa-Itoh, Eiki Yamashita, Kazumasa Muramoto, Tomitake Tsukihara, Shinya Yoshikawa

**Affiliations:** From the ‡ Picobiology Institute and; ¶ Department of Life Science, Graduate School of Life Science, University of Hyogo, 3-2-1 Koto, Kamigori, Akoh, Hyogo 678-1297,; the § Institute for Protein Research, Osaka University, 3-2 Yamadaoka, Suita, Osaka 565-0871, and; the ‖ Japan Science and Technology Agency, CREST, 4-1-8 Honcho, Kawaguchi, Saitama 332-0012, Japan

**Keywords:** enzyme mechanism, cytochrome c oxidase (complex IV), proton pump, mitochondrial membrane potential, X-ray crystallography, bioenergetics, heme, metalloenzyme, azide, copper

## Abstract

Cytochrome *c* oxidase (CcO) is the terminal oxidase of cellular respiration, reducing O_2_ to water and pumping protons. X-ray structural features have suggested that CcO pumps protons via a mechanism involving electrostatic repulsions between pumping protons in the hydrogen-bond network of a proton-conducting pathway (the H-pathway) and net positive charges created upon oxidation of an iron site, heme *a* (Fe*_a_*^2+^), for reduction of O_2_ at another iron site, heme *a*_3_ (Fe*_a_*_3_^2+^). The protons for pumping are transferred to the hydrogen-bond network from the N-side via the water channel of the H-pathway. Back-leakage of protons to the N-side is thought to be blocked by closure of the water channel. To experimentally test this, we examined X-ray structures of the azide-bound, oxidized bovine CcO and found that an azide derivative (N_3_^−^–Fe*_a_*_3_^3+^, Cu_B_^2+^–N_3_^−^) induces a translational movement of the heme *a*_3_ plane. This was accompanied by opening of the water channel, revealing that Fe*_a_*_3_ and the H-pathway are tightly coupled. The channel opening in the oxidized state is likely to induce back-leakage of pumping protons, which lowers the proton level in the hydrogen-bond network during enzymatic turnover. The proton level decrease weakens the electron affinity of Fe*_a_*, if Fe*_a_* electrostatically interacts with protons in the hydrogen-bond network. The previously reported azide-induced redox-potential decrease in Fe*_a_* supports existence of the electrostatic interaction. In summary, our results indicate that the H-pathway is critical for CcO's proton-pumping function.

## Introduction

**Figure 1.**
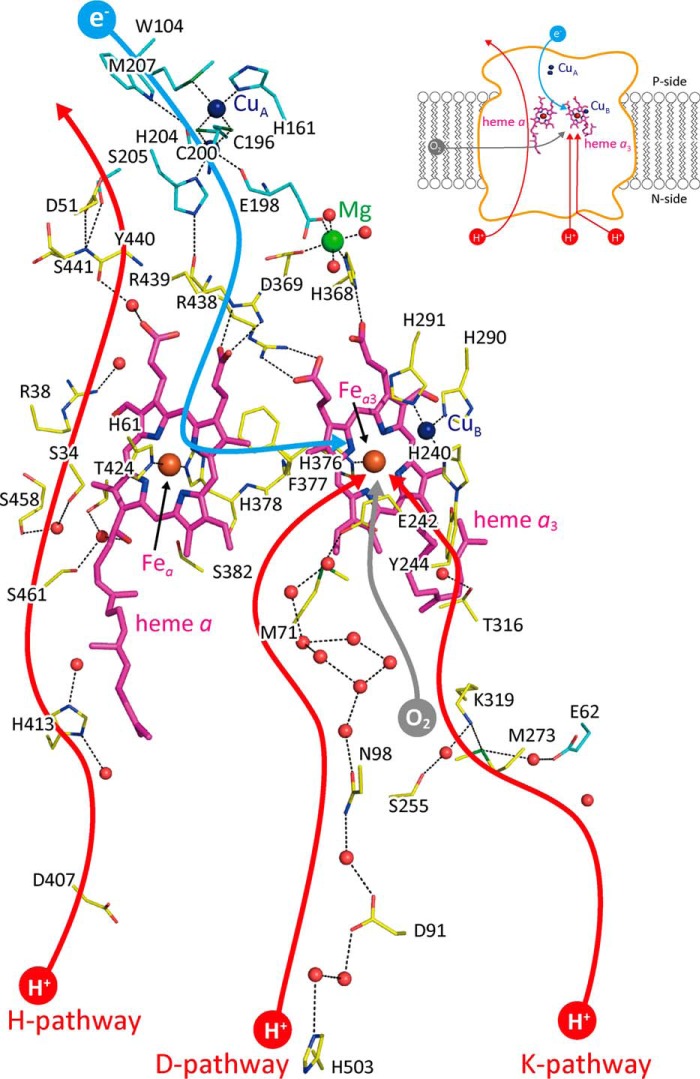
**X-ray structure of bovine heart CcO.** The locations of the iron, copper, and magnesium atoms are shown by *reddish brown*, *dark blue*, and *green balls*, respectively. *Small red balls* are located at the positions of water molecules. *Magenta* structures denote hemes *a* and *a*_3_ as labeled. The positions of the central iron atoms, Fe*_a_* and Fe*_a_*_3_, of the hemes shown in *reddish brown balls* are indicated by *arrows*. The *dark blue*, *red,* and *green* portions of amino acids denote nitrogen, oxygen, and sulfur atoms, respectively. The carbon atoms of amino acid residues of subunits I and II are labeled by *yellow* and *blue sticks*, respectively. The possible pathways for O_2_, electrons, and protons for making water molecules and for pumping are denoted by *gray, blue,* and *red curves*, each with an *arrowhead.* The structure and location of the water exit pathway are still under debate. The *inset* is a schematic representation of the locations of the redox-active metal sites and the pathways for transportation of the substrates (O_2_, electrons, and protons) within the overall CcO structure. This figure is based on the X-ray diffraction data given in PDB 5B1A.

Cytochrome *c* oxidase (CcO) ^5^ is the terminal oxidase of cellular respiration that reduces O_2_ to water, coupled with a proton-pumping process. The enzyme includes two redox-active metal sites as the low-potential sites (heme *a* (Fe*_a_*) and Cu_A_). These sites donate electrons to the O_2_ reduction site that comprises two high potential metal sites (heme *a*_3_ (Fe*_a_*_3_) and Cu_B_) (1, 2). Two proton-conducting pathways, designated as the K- and D-pathways as shown in Fig. 1, are located in the protein for connecting the O_2_ reduction site with the N-side surface of the CcO molecule. These pathways transfer protons for making water molecules after (or coupled with) donation of electrons to the O_2_ reduction site. The third proton-conducting pathway, the H-pathway, extends for proton pumping from the N-side surface to the P-side surface.

Extensive X-ray structural analyses of the system strongly suggest that proton pumping is driven by electrostatic repulsions between the protons transferred from the N-side by hydronium ions through a water channel, which forms part of the proton pumping pathway (the H-pathway) and the net positive charges created upon oxidation of heme *a* for reduction of O_2_ bound to heme *a*_3_. The directionality of the proton pump is provided by closure of the water channel after the pumping protons are collected (1, 3, 4). The water molecules (or the hydronium ions) are driven through the water channel by thermal motion of the protein moiety. Water cavities, where at least one mobile water molecule is stored, are located within the water channel. These water cavities accelerate the water exchange between the N-side and the hydrogen-bond network in the H-pathway that extends to the P-side, because the water molecules in the water cavities are mobile without significant interaction with the protein moiety. Extensive X-ray structural analyses thus far show that the water channel is closed by elimination of the largest cavity near the junction point with the hydrogen-bond network. The water channel opens only when the O_2_ reduction site comprising Fe*_a_*_3_ and Cu_B_ is in the ligand-free fully reduced state and is closed in other oxidation states or in strong ligand (such as CO, NO and O_2_)-bound fully reduced states (5).

In contrast, proton-pumping mechanisms without involvement of the H-pathway have been proposed, based on mutational analyses of bacterial CcOs. Bovine CcO includes well-conserved residues, Glu^242^ and Asn^98^, near the upper end and halfway in the D-pathway, respectively, as described in Fig. 1. Bacterial E242Q mutant CcOs show neither the O_2_ reduction nor proton pumping, whereas bacterial N98D mutant CcOs reduce O_2_ without proton pumping. Based on these mutational results, it has been proposed that Gln^242^ abolishes transfer of protons for both pumping and O_2_ reduction, whereas Asp^98^ blocks only the proton-pumping transfer, at least, in bacterial CcOs (1–3). However, D51N mutation for bovine heart CcO abolishes proton-pumping function without decreasing the O_2_ reduction activity, confirming the critical role of the H-pathway in proton pumping of bovine CcO (6, 7), whereas Asp^51^ is not conserved in bacterial CcOs, and any mutation for the bacterial H-pathway residues shows no influence to both O_2_ reduction and pumping activities. One of the simplest interpretations for these mutational analysis results is that the mechanism of proton pumping of CcO is not completely conserved.

The proton/electron coupling mechanism of bovine CcO, proposed based on the static X-ray structural results, has been confirmed by mutational analyses of the function of the H-pathway, as described above (6, 7). However, the proposal has not been confirmed by experimental (or functional) analyses of the coupling process in the enzyme. For example, the X-ray structure of the hydrogen-bond network of the H-pathway, which is attached to heme *a* peripheral groups, strongly suggests that the electrostatic interactions between positively charged heme *a* and protons on the hydrogen-bond network drive proton-active transport (1–3, 7). However, the X-ray structural results themselves do not clearly show the existence of the electrostatic interactions sufficiently strong to promote active proton transfer. Thus, it is desirable to examine the effects of structural perturbation for the H-pathway on the enzymatic function of the bovine CcO.

Since the pioneering studies on respiration by Keilin and Hartree 78 years ago (8), azide has been known to be a potent inhibitor of CcO, which is nearly as strong as cyanide at low pH (9, 10). It was recognized that this inhibitor, which is obviously chemically different from O_2_, could be an excellent probe for investigating the function of the O_2_ reduction site of the enzyme. Extensive investigations of the interactions between azide and CcO are fairly limited, compared with investigations using CO and cyanide. One of the reasons is that the azide-induced absorption spectral changes of the oxidized CcO, as isolated from bovine heart, are very weak, and as a result, consistency among the reported results from different groups, which likely used different enzyme preparations, has tended to be fairly low (11–14).

All of the redox-active sites of the oxidized CcO as isolated from bovine heart are in the oxidized state (1, 15). Its reactivity with cyanide and its electron transfer and proton-pumping functions indicate that the fully oxidized form (or the “as prepared” form) is not directly involved in catalytic turnover (1). Thus, the preparation is designated as “resting oxidized” CcO. Although the physiological significance of this form remains controversial, it has been the most extensively investigated among the various forms and derivatives of CcO because of the availability of samples with high purity and yield (1, 2). Recently, it has been established that the resting oxidized form has a peroxide moiety bridged between Fe*_a_*_3_^3+^ and Cu_B_^2+^ (16).

Recently, spectral changes in the absorption, which occur with binding of azide to the resting oxidized CcO, have been well-established using the CcO preparation purified by recrystallization. This investigation identifies a two-step binding of azide to the O_2_ reduction site revealed by accurately measuring the small spectral changes showing that CcO has two azide-binding sites with high and low affinities (*K_d_* values of 78 μm and 42 mm, respectively) (17). Resonance Raman analyses showed that bound peroxide is removed by the initial azide binding to Fe*_a_*_3_^3+^ without influencing a spin state of heme *a*_3_. The absence of a spin-state transition provides much smaller spectral changes in absorbance upon binding of azide compared with the spectral changes that occur with cyanide binding accompanied by a high- to low-spin state transition (17, 18).

The IR analyses of azide binding to the enzyme in the five different overall oxidation states (*i.e.* 0 (resting oxidized) to the 4-electron reduced (the fully reduced) states) indicate that, in each oxidation state except for the fully reduced state, CcO shows sharp azide bands assignable to Fe*_a_*_3_^3+^–N_3_^−^ or Cu_B_^2+^–N_3_^−^, depending on the overall oxidation state. Based on these results, it has been proposed that the two metal sites in the O_2_ reduction site are occupied by azide simultaneously (12). However, the IR results do not preclude the possibility that a single azide molecule binds to either one of the two metal sites in the O_2_ reduction site. The X-ray structure of the azide-bound resting oxidized CcO has been determined at 2.9 Å resolution (PDB code 1OCZ). This structure shows a bridging ligand between the two metals in the O_2_ reduction site. This is inconsistent with the two sharp azide IR bands, which suggest terminal azide binding to a metal ion (19, 20). However, binding of single azide ion to the O_2_ reduction site is consistent with the narrow ligand binding space of the O_2_ reduction site. In fact, an early careful titration analysis indicates stoichiometric binding of a single azide ion to the resting oxidized bovine CcO (10). Furthermore, X-ray structural results thus far suggest that the site has the capacity to bind only a single diatomic molecule or ion, such as O_2_, CO, NO, and CN^−^ (1).

To gain insights into the proton-pumping function of CcO as well as to clarify the structural basis of the inconsistent observations with regard to binding of azide to CcO, we reexamined the X-ray structure of the azide-bound resting oxidized CcO at significantly improved resolution (∼1.85 Å). The present results reveal that the azide-binding sites, Fe*_a_*_3_^3+^ and Cu_B_^2+^, have an unexpectedly high tolerance for large ligands and that each can accept a terminal azide ion. The binding of two azide ions to the two metal centers induces opening of the water channel without changing the oxidation and spin states of the metal centers, which is uniquely detectable in the azide-bound state. The channel opening is highly likely to disturb the system for blockage of the water channel against the pumping proton back leaks to impair the normal enzymatic function, which is consistent with the proposal that the H-pathway is involved critically in the proton-pumping function.

## Results

### Two terminal azide-binding modes at 20 mm azide

**Figure 2.**
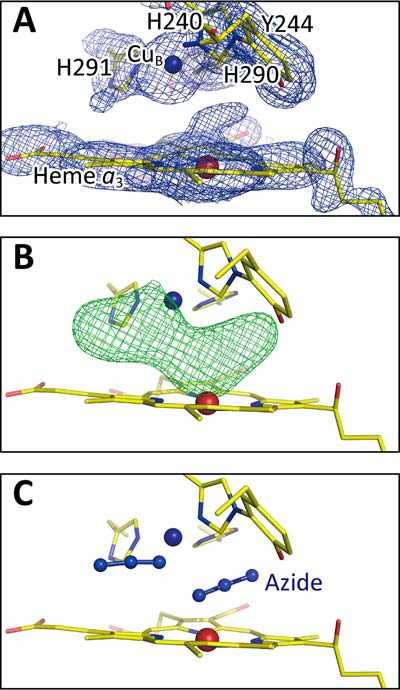
**Electron density maps of the O_2_ reduction site of bovine heart resting oxidized CcO obtained from the crystals exposed to 20 mm azide at pH 5.7 for 4 days.** *A*, MR/DM map of the O_2_ reduction site. *B* and *C*, the *F_o_* − *F_c_* map without and with azide ions, respectively, showing the residual densities (*green mesh*) at +3.0 σ. Significant residual density is not detectable in *C.*

The azide binding was confirmed by the visible spectral changes in the vicinity of the 650 nm shoulder as described previously (17). Fig. 2, *A* and *B,* shows the electron density maps of the O_2_ reduction site for the MR/DM map (see “X-ray structure determinations and refinements” under “Experimental procedures”) and the *F_o_* − *F_c_* map without azide ions, respectively, at 1.85 Å resolution. The structural refinement converged well for two azide ions, each coordinating to respective Fe*_a_*_3_ and Cu_B_, at the full occupancy, and the *F_o_* − *F_c_* map gave no significant residual electron density between the two metals (Fig. 2*C*). It is noteworthy that no other technique other than high-resolution X-ray crystallography is able to reveal the truly unexpected geometry of azide binding.

**Figure 3.**
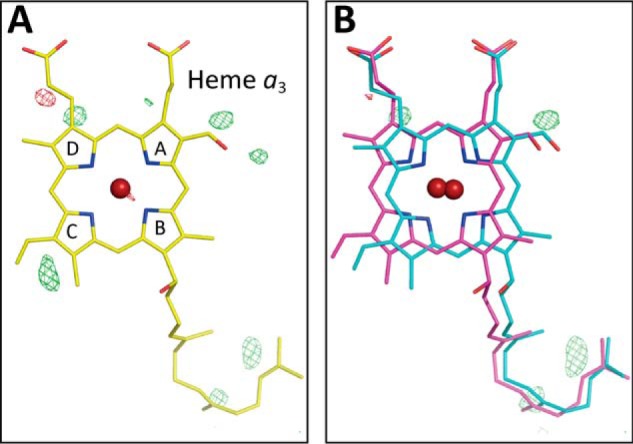
**X-ray structure of heme *a*_3_ in the presence of 20 mm azide.** *A*,*F_o_* − *F_c_* map determined assuming a single structure of heme *a*_3_. The residual densities are indicated with *green mesh* at +3.0 σ, and the converged structure is shown. *B*, *F_o_* − *F_c_* maps assuming the structures A and B of heme *a*_3_ (indicated by *magenta* and *light blue sticks*, respectively) in the 1:1 occupancy ratio. No significant residual density is detectable at the +3.0 σ level near the ring C.

Whereas the refinement resulted in abnormal *B*-factors of heme *a*_3_, of which the averaged value is significantly higher than that of heme *a* (28.1 Å^2^ *versus* 23.3 Å^2^), in the *F_o_* − *F_c_* map, significant residual densities are detectable near ring C (in the PDB naming scheme) as shown in Fig. 3*A*. These results strongly suggest the existence of multiple structures. The two structures shown in Fig. 3*B* in equal occupancy with each other essentially eliminate the residual densities detectable near ring C in Fig. 3*A* and provide approximately equal *B*-factors (25.2 and 23.3 Å^2^, respectively) closely similar to that of heme *a* (23.3 Å^2^). The *B*-factors for structures A and B, calculated assuming the occupancy ratio of 0.4/0.6, are 23.1 and 25.1 Å^2^, respectively, which are also closely similar to that of heme *a*, as given in Table 1. The monotonous increase and decrease in the *B*-factors of structures A and B, respectively, with the occupancy increase in structure A (Table 1) indicate that a *B*-factor, 24.2 Å^2^, for both structures is attained at the occupancy ratio of 0.45:0.55. Thus, the reliability of the assessment of the occupancy ratio based on the average *B*-factors is higher than ±0.1. The two structures indicate a translational shift of the heme plane fixing the two propionate carboxyl groups and the terminal end of the hydroxy farnesyl ethyl group (Fig. 3*B*). The converged structure, assuming a single structure shown in Fig. 3*A,* is located between the two structures. The location of one of the structures (Fig. 3*A*, shown in *magenta*) is close to that of the resting oxidized form. In this paper, the azide-induced structure similar to that of the resting oxidized state is designated as structure A, and the other structure is designated as structure B throughout. The heme plane shift between the two structures (from *magenta* to *light blue*) is greater than the structural changes that occur upon complete reduction of resting oxidized CcO as given in Fig. 3*B*, although the change is in the same direction.

**Table 1:**
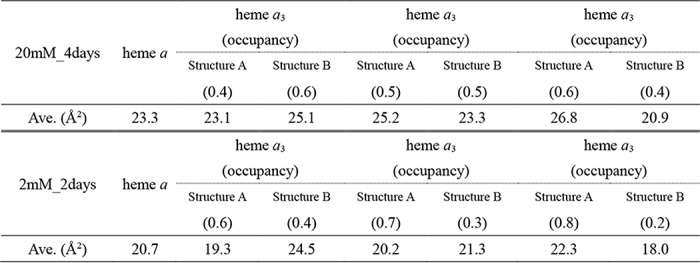
**Effects of structure occupancy on the B-factor of heme *a*_3_** Azide concentrations and exposure periods are indicated with the term “XX mM_Ydays.” For structure A, the nonshifted conformation is closely similar to the conformation of the resting oxidized state; structure B is the azide-induced shifted conformation. The averaged *B*-factors were calculated using the atoms of the composing porphyrin ring of heme *a* or heme *a*_3_.

**Figure 4.**
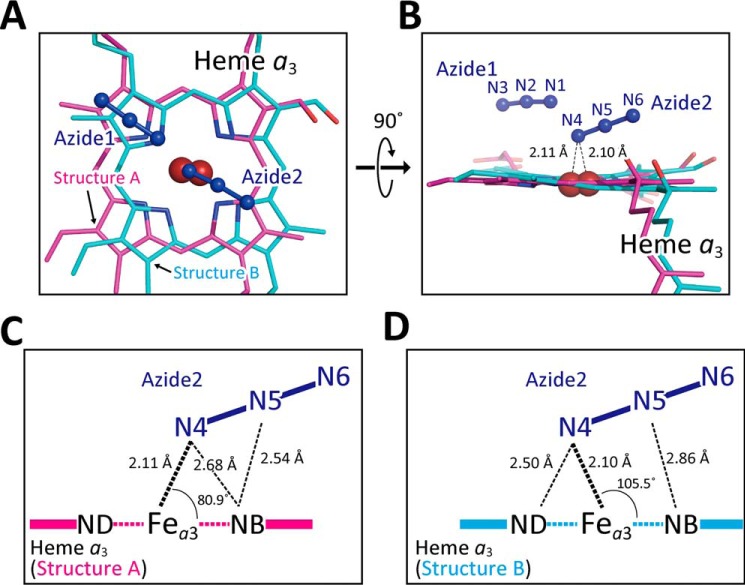
**Two modes of terminal azide bindings to Fe*_a_*_3_ at 20 mm azide.** *A* and *B*, atomic model of terminally-bound azide ions. Azide 1 and azide 2 in *dark blue* indicate azide ions bound to Cu_B_ and heme *a*_3_, respectively. Heme *a*_3_ planes giving structures A and B are drawn in *magenta* and *light blue*, respectively. *C* and *D*, geometry of bindings of azide 2 giving structures A and B, respectively, in the same color-code as in *A* and *B*.

As shown in Fig. 4, *A* and *B*, azide 1 interacts with both pyrrole rings D of structure A and structure B with distances ∼3.5 and ∼3.3 Å, respectively. The geometries of azide 1 are summarized in Table 2. The strongest interaction of the terminally-bound azides to CcO is the coordination bond between Fe*_a_*_3_ and one of the terminal nitrogen atoms of azide 2. The N–Fe–N angles of structure A and structure B are 80.9 and 105.5°, respectively. Furthermore, the terminal and central nitrogen atoms have close contacts with nitrogen atoms of pyrrole B of structure A with distances of 2.68 and 2.54 Å, respectively (Fig. 4*C*), and with those of pyrrole rings of B and D of structure B, with distances of 2.86 and 2.50 Å, respectively (Fig. 4*D*). The heme shift between structures A and B is accompanied by distortion of the Fe*_a_*_3_–N coordination, which induces the multiple conformations in helix X as described below.

**Table 2:**
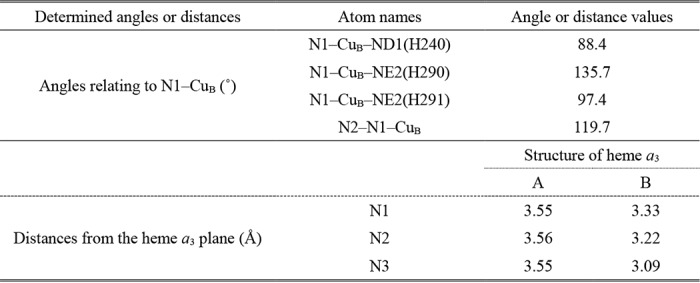
**Geometries of an azides bound to Cu_B_, in the crystal equilibrated with 20 mm azide solution** Letters A and B indicate the structures A and B of heme *a*_3_, respectively. Other atomic names are given in Fig. 3. Heme *a*_3_ plane was calculated on the basis of FE, NA, NB, NC, and ND atoms in each heme *a*_3_ structure by geomcalc program (38).

### Azide-induced conformational changes in helix X at 20 mm azide

**Figure 5.**
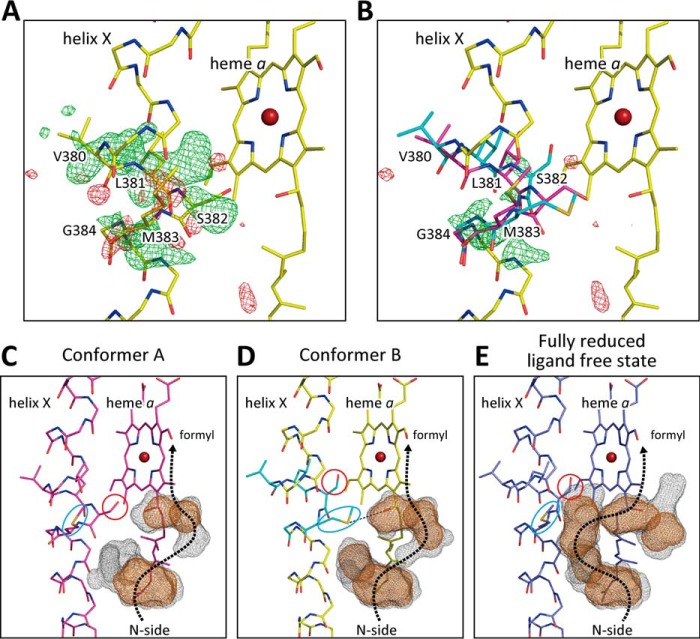
***F_o_* − *F_c_* maps of a segment (from Val^380^ to Gly^384^) of helix X.** The residual electron densities are indicated with *green mesh* (+3.0 σ) and *red mesh* (−3.0 σ). *A*, determined assuming the conformation of the resting oxidized state (*yellow*). *B*, determined assuming the two conformations, one closely similar to that of the resting oxidized state (*magenta*) (conformer A) and the other obtained with 20 mm azide (*light blue*) (conformer B), in 1:1 occupancy ratio. *C* and *D*, water cavities located near the P-side end of the water channel in conformers A and B, detectable at 20 mm azide, respectively. The *brown-* and *gray-dotted surfaces* are determined by the program VOIDOO (39) using probes with a radius of 1.2 and 0.8 Å, respectively. *E*, cavities of the open conformation of the fully reduced ligand-free state, detectable in areas corresponding to those of *C* and *D*. The same color code as *C* and *D* was used to indicate the water-accessible surfaces. The *dotted arrows* denote possible locations of water channels. The locations of side chains of Ser^382^ and Met^383^ are indicated by *red circles* and *blue ovals*, respectively. The hydrogen bond between Met^383^ and the OH group of the hydroxy farnesyl ethyl group of heme *a* is indicated by a *dotted line*.

The structural refinement for helix X with the conformation of the resting oxidized CcO, in which the water channel is closed, provides higher average *B*-factors of amino acid residues from 380 to 384 of the helix X (∼35 Å^2^) than those of the other residues of the helix (∼25 Å^2^). The *F_o_* − *F_c_* map provides significant positive and negative residual densities (Fig. 5*A*), suggesting a multiple conformation in the helix X closely similar to those observable when the open/closed conformation change in the water channel is induced upon ligand binding to the fully reduced ligand-free state or oxidation state changes of the fully reduced ligand-free state (5). Structural refinements were performed for two conformers of helix X with different occupancy ratios, each at 0.1 step. The multiple conformation with the two structures in equal occupancy (0.5:0.5) provides two conformers with closely similar values of *B*-factor as shown in Table 3 and the lowest residual electron densities among those obtained assuming various occupancy ratios (Fig. 5*B*).

**Table 3:** **Effects of conformer occupancy on the *B*-factor of flexible amino acid residues in helix X at 20 mm azide** Conformer A is closely similar to the resting oxidized state. Conformer B is an azide-induced open conformation.

Conformer	Occupancy	Val-380	Leu-381	Ser-382	Met-383	Gly-384	Average
A	0.4	21.0	19.5	22.8	23.8	25.7	22.6
B	0.6	30.7	30.0	32.0	29.6	28.0	30.1
A	0.5	24.7	23.3	27.6	28.2	27.1	26.2
B	0.5	27.3	25.5	28.7	25.8	26.7	26.8
A	0.6	27.9	26.6	31.9	32.3	28.3	29.4
B	0.4	23.4	20.7	24.5	21.6	24.8	23.0

One of the conformers (conformer A) is closely similar to that of the closed conformation, detectable in the resting oxidized state, in both of the main and side chains (Fig. 5*C*). The main-chain conformation of the other conformer (conformer B) (Fig. 5*D*) is closely similar to that of the open conformer of the ligand-free fully reduced state (Fig. 5*E*), whereas the side chain of Met^383^ (indicated with a *light blue oval* in Fig. 5*D*) folds in a bent conformation, definitely different from that of the open conformation of the ligand-free fully reduced state. This bent conformation is somewhat unusual and gives significantly higher *B*-factors compared with those of nearby residues. The unusual conformation of the Met^383^ side chain appears to be stabilized by a hydrogen bond formation with the hydroxyl group of the hydroxy farnesyl ethyl group of heme *a*, as marked by a *dotted line* in Fig. 5*D*. As shown in Fig. 5*D*, the side chain of Met^383^ eliminates one of the large cavities shown with *brown cages* detectable in the open conformation of the ligand-free fully reduced state (Fig. 5*E*). The position of the side chain of Ser^382^ marked by a *red circle* (Fig. 5*D*) is essentially identical to its position in the open conformation (Fig. 5*E*).

As shown in Fig. 5*E*, in the open conformation detectable in the ligand-free fully reduced state, the five water cavities located in the water channel near the junction point with the hydrogen-bond network (or the formyl group of heme *a*) contribute significantly to the accessibility of the water molecules from the N-side through the water channel, the location of which is marked by a *dotted arrow* in Fig. 5*E*. In conformer A, the side chain of Ser^382^, indicated with a *red circle* in Fig. 5*C*, eliminates two of the five cavities detectable in the ligand-free fully reduced state to essentially block the water channel as shown in Fig. 5*C*. In this conformation, an alternative water channel along the shortest distance between the two cavities near heme *a* and the one located closer to the N-side could be used as indicated with a *dotted arrow* in Fig. 5*C*. However, water exchange between these cavities is highly unlikely to occur at least within the physiological time scale (about 1 ms for the single turnover of the CcO reaction (1)), because even the water-accessible surfaces calculated with a probe radius of 0.8 Å (indicated by the *gray-dotted surfaces*) have no direct contact between these cavities. Thus, this conformation is designated as the “closed conformation.” This conformer is identical to the conformer observable in resting oxidized CcO within experimental accuracy.

The water-exchange efficiency difference between conformers A and B is obvious when comparing the water access surfaces calculated using a probe radius of 0.8 Å, as indicated by the gray-dotted surfaces. In conformer B, the water-accessibility surfaces of the two water cavities near heme *a* are more closely located with each other (Fig. 5*D*) compared with the corresponding surfaces in conformer A (Fig. 5*C*). Thus, the structure suggests that conformer B is unlikely to close the water channel as tightly as conformer A does. The conformation is designated as the azide-induced open conformation. Although Met^383^ eliminates the water cavity detectable in the open conformation (observable in the fully reduced ligand-free state (Fig. 5*E*)), the heme *a*_3_ migration induced by terminally-bound azide ions creates an alternative pathway for water exchange (Fig. 5*D*). Thus, 20 mm azide induces the closed/open conformational transition without changing the oxidation state of the O_2_ reduction site.

### X-ray structure of the azide-bound oxidized CcO at 2 mm azide

**Figure 6.**
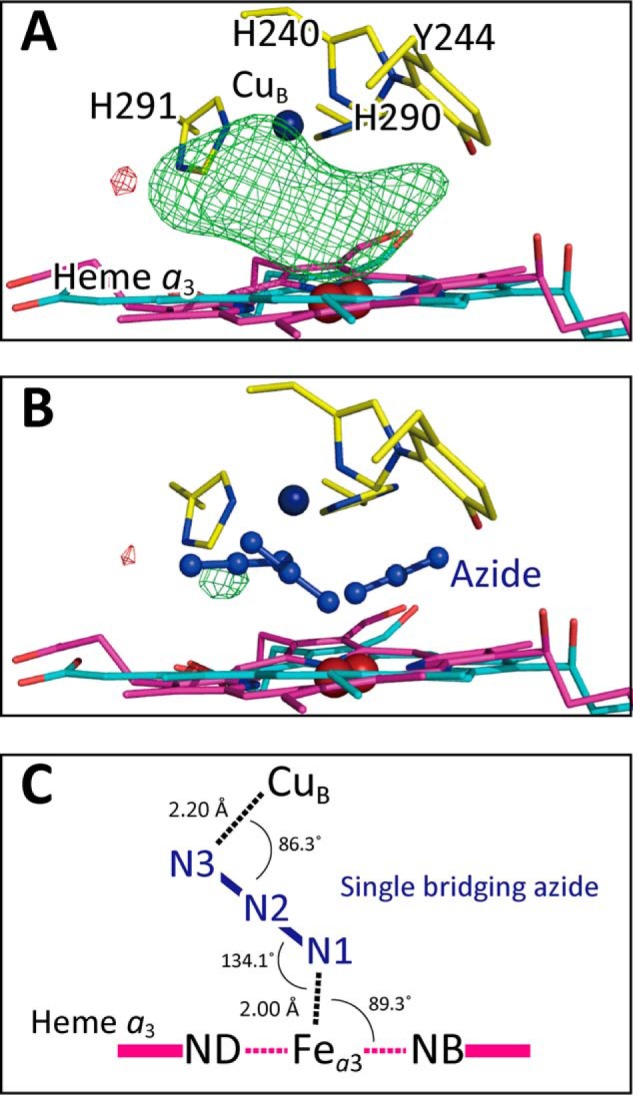
***F_o_* − *F_c_* maps of the O_2_ reduction site of bovine heart resting oxidized CcO obtained from crystals exposed to 2 mm azide.** *A*, *F_o_* − *F_c_* map without azide ion, showing the residual densities (*green mesh*) at +3.0 σ identical to the σ level of Fig. 2*B*, *B*, *F_o_* − *F_c_* maps with two terminally-bound azide ions at Fe*_a_*_3_ and Cu_B_, each at 30% occupancy, and a single bridged azide ion, giving essentially no residual density, *C*, schematic representation of the geometry of the single bridging azide.

Effects of decreasing the azide concentration on the X-ray structure of the azide derivatives were analyzed for investigation of the mechanism of the two-step binding of azide to CcO revealed by spectroscopic analyses as described above (17). As shown in Fig. 6*A*, the *F_o_* − *F_c_* electron density at 2 mm azide at the σ level identical to that of Fig. 2*B* (the *F_o_* − *F_c_* electron density at 20 mm azide) indicates clear electron density with higher density at the central part than in the case of 20 mm azide. Structural refinement for a single bridging azide ion coexisting with the two partially occupied azide ions, each of which is terminally-bound as at 20 mm azide, in the occupancies of 0.7 (single) and 0.3 (double) was converged well and resulted in the lowest residual density in the *F_o_* − *F_c_* map as shown in Fig. 6*B*. Effects of the occupancy ratio on the average *B*-factor of the bound azides confirm that the above occupancy ratio provides the lowest and similar values of *B*-factor for the two structures as given in Table 4. The geometries of the azide bridging between Fe*_a_*_3_ and Cu_B_ are given in Fig. 6*C*, which are consistent with the previously reported structure of azide derivatives prepared at the azide concentration significantly lower than 2 mm (19). Binding of three azide ions simultaneously to the O_2_ reduction site is not possible due to steric constraint. The two metal sites in the two azide-bound states do not have sufficient space to accept an additional single azide ion bridging between the two metals.

**Table 4:** **Effects of azide occupancy on the averaged *B*-factor of nitrogen atoms of azide ion at 2 mm**

Azide name	Occupancy	Averaged *B*-factor
		Å*^2^*
Single bridging azide	0.9	26.8
Terminal binding azide	0.1	7.5
Single bridging azide	0.8	26.1
Terminal binding azide	0.2	15.6
Single bridging azide	0.7	24.3
Terminal binding azide	0.3	22.1
Single bridging azide	0.6	21.8
Terminal binding azide	0.4	25.2
Single bridging azide	0.5	19.7
Terminal binding azide	0.5	29.6

The structure of heme *a*_3_ at 2 mm azide determined assuming a single structure also shows an average *B*-factor higher than that of heme *a* and residual densities near ring C, suggesting a multiple structure. The refinement assuming two conformations with the same procedures as used for the analysis of the 20 mm azide structure provides two structures at 0.7/0.3 occupancy ratio. The dominant (0.7) and minor (0.3) structures are closely similar to those of structures A and B, observed at 20 mm azide, respectively (*i.e.* the nonshifted structure essentially identical to that of the resting oxidized CcO and the azide-induced shifted structure, respectively). Similarly, the helix X structure near Ser^382^ at 2 mm azide indicates coexistence of conformers A and B in a ratio of 0.8/0.2 as given in Table 5. If the state with two terminally-bound azide ions provides 1:1 multiple structures (structures A and B in heme *a*_3_ and conformers A and B in helix X as at 20 mm azide) and if the single azide bridging species provides essentially identical structures to those of the structure A in heme *a*_3_ and conformer A in helix X, the ratio for the single azide-bound state/the two azide-bound state of 7:3 provides the ratio of 8.5:1.5 for structures A to B in heme *a*_3_ and conformers A to B in helix X. The experimental results given in Table 5 for helix X and heme *a*_3_ structure (8:2 and 7:3, respectively) are consistent with the expected value within experimental error. Thus, the results suggest that the two azide ions terminally bound to Fe*_a_*_3_ and Cu_B_ in the presence of 2 mm azide provide the two structures in both heme *a*_3_ and helix X as the two terminally-bound azide ions do at 20 mm.

**Table 5:** **Effects of azide on X-ray structures of resting oxidized CcO** The values indicate the occupancy of each multiple structure.

N_3_^−^ concentration	Exposure	O_2_ reduction site		Heme *a*_3_		Helix X		PDB ID
		1 N_3_^−^	2 N_3_^−^	Structure A (nonshifted)	Structure B (shifted)	Conformer A (closed)	Conformer B (open)	
20 mm	4 days	0.0	1.0	0.5	0.5	0.5	0.5	5Z84
	3 days	0.0	1.0	0.5	0.5	0.5	0.5	5Z86
	2 days	0.4	0.6	0.4	0.6	0.5	0.5	5ZCP
	2 days*^a^*	0.0	1.0	0.5	0.5	0.5	0.5	5Z85
2 mm	2 days*^a^*	0.7	0.3	0.7	0.3	0.8	0.2	5ZCO

*^a^* Different batches of crystals were used for these experiments.

### Hydrogen-bond structures between Tyr^244^ and the OH group of the hydroxy farnesyl ethyl group of heme a_3_

**Figure 7.**
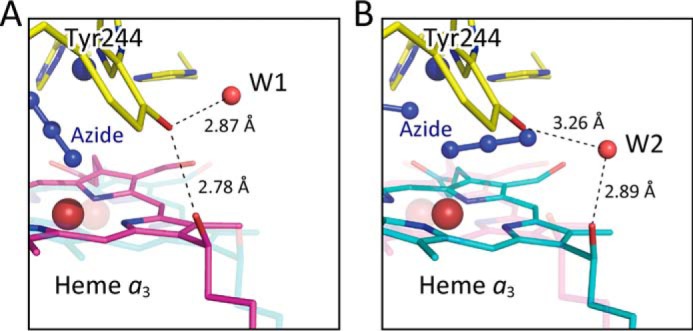
**Hydrogen bond system between the hydroxy farnesyl ethyl group and Tyr^244^ covalently linked to one of the imidazoles coordinated to Cu_B_ at 2 mm azide.** *A*, one of the structures detectable at 2 mm azide, closely similar to that detectable in the resting oxidized state showing the direct hydrogen bonding between the two OH groups of Tyr^244^ and hydroxy farnesyl ethyl group (structure A). The Tyr^244^–OH group has a fixed water molecule (*W1*) hydrogen-bonded. *B*, (structure B) another structure detectable at 2 mm azide showing a large translational shift of heme *a*_3_ plane from that of structure A. The direct hydrogen bond between the two OH groups is replaced by a bridging water molecule (*W2*) hydrogen-bonded to the two OH groups.

In structure A of heme *a*_3_ at 2 mm azide (Fig. 7*A*), a hydrogen bond between Tyr^244^–OH and the OH group of the hydroxy farnesyl ethyl group of heme *a*_3_ is detectable, and a water molecule, W1, is hydrogen-bonded to Tyr^244^–OH. The structure is closely similar to that in the resting oxidized state in the absence of azide. The hydrogen bond is cleaved by the azide-induced heme *a*_3_ plane shift to introduce a water molecule, W2, in structure B, as shown in Fig. 7*B*. The occupancies of W1 and W2 at 2 mm azide, estimated with the procedure described under “Experimental procedures,” are 30 and 25%, respectively. The former is nearly identical to that of W1 detectable in the resting oxidized CcO in the absence of azide (about 30%). In contrast, the interactions of heme *a*_3_ with Tyr^244^ in structures A and B at 20 mm azide are essentially identical to those at 2 mm azide as given in Fig. 7. However, the occupancy of W2 is 65%, whereas W1 is undetectable at the 3 σ level. Therefore, the microenvironment of Tyr^244^ OH group at 2 mm azide, which receives W1 at 30% occupancy (Fig. 7*A*), is different from that in structure A at 20 mm azide, in which no significant W1 is detectable.

The azide-induced hydrogen bond break as given in Fig. 7 is not detectable with the heme *a*_3_ shift induced by reduction of heme *a*_3_ of bovine CcO (21). However, it has been reported that reduction of fully oxidized bacterial CcO induces loss of a hydrogen bond, which is very similar to the hydrogen bond loss observed upon addition of a high concentration of azide to the resting oxidized bovine CcO (22).

### Saturation of the terminal azide-binding sites in the resting oxidized CcO crystals

The two azide terminally-bound CcO crystals were prepared by exposing resting oxidized CcO crystals to 20 mm azide for 3 days at 4 °C as described under “Experimental procedures.” Complete saturation of the azide-binding metal sites is not attained by exposure of CcO to 20 mm azide for 2 days, whereas elongation of the exposure time up to 4 days does not significantly change the X-ray crystal structures (Table 5). Thus, in contrast to the binding kinetics for the second (low affinity) azide-binding site in solution (faster than the time scale of minutes) (17), 3 days of exposure at 4 °C is necessary for saturation of the two terminal azide-binding sites. The discrepancy is likely to be due to restriction of flexibility in the O_2_ transfer pathway (23) induced by the crystal packing. As typically shown in the results obtained by a 2-day exposure to 20 mm azide (Table 5), the process of azide binding to the metal sites does not tightly synchronize with the progress of the structural changes in the heme *a*_3_ and helix X. The azide saturation period was found to be somewhat dependent on the CcO preparation batch. Some CcO batches provided the crystals, which require 2 days for saturation of the two terminal azide-binding sites (Table 5).

Even at the highest azide concentration employed (20 mm) in this work, no azide ion was detectable on the transmembrane surface of the CcO molecule where azide binding has been proposed based on the X-ray structure of the azide-bound bovine heart CcO at 2.9 Å resolution (19). In the present structure, a fairly stable fatty acid tail of a triacylglycerol molecule occupies the electron density previously assigned to an azide ion.

## Discussion

### Reactions of azide with the resting oxidized CcO

The present results indicate that there is unexpectedly high flexibility in the O_2_ reduction site that allows the site to accept two triatomic ligands. The reported two terminal azide IR bands (12) are likely due to the two terminally-bound azide ions in the present X-ray structure.

The azide ion bridging between the two metals in the O_2_ reduction site detectable in the X-ray structure at 2 mm azide does not provide any detectable band in the azide stretch band region (2100–2000 cm^−1^). The results suggest that bridging between the two metals decreases the polarization of the azide molecule significantly, which weakens the IR band intensity. The *B*-factors of nitrogen atoms of the azide ion bridging between the two metals in the present X-ray structures are roughly identical with those of the terminally-bound azide ions. Thus, the bridging between the two metals does not increase the diversity of orientation of the bound azide. (The diversity increase in orientation of metal-bound ligand broadens the IR band to weaken the maximal band intensity.) Thus, we propose that the main reason for weakening of the azide band is the polarity decrease upon bridging between the two metals.

The terminal binding of two azide ions simultaneously to Fe*_a_*_3_ and Cu_B_ was observed also at 2 mm azide as described above, and the occupancies of the two terminally-bound azide ions are identical to each other irrespective of the azide concentration down to concentrations that are insufficient to saturate the two terminal binding sites. Thus, the terminally-bound azide at Fe*_a_*_3_^3+^ is as stable as the terminally-bound azide at Cu_B_^2+^. Alternatively, it is possible that any single azide terminally bound at either one of the two metals is much less stable than the azide bridging between the two metals.

Earlier extensive absorption spectral analysis for azide binding to the resting oxidized CcO (17) reveals a two-step absorbance change with an increase in the azide concentration, in which each step is induced by a single azide ion. The high-affinity binding eliminates a resonance Raman band assignable to the O–O stretch band of the peroxide bound to the resting oxidized CcO, leaving a band assignable to the ν_16_ porphyrin mode of the high-spin heme *a*_3_. This porphyrin mode band is not influenced by increasing the azide concentration up to 100 mm sufficient for saturating the low-affinity (second) azide-binding site. Based on these spectral results, it has been proposed that the high-affinity binding terminally to Fe*_a_*_3_^3+^ eliminates the peroxide bridging between Fe*_a_*_3_^3+^ and Cu_B_^2+^ in the resting oxidized CcO, whereas both Fe*_a_*_3_^3+^ and Cu_B_^2+^ sites are fully occupied by terminally-bound azide ions at high-azide concentrations (17).

The present X-ray structural results show that the high-affinity azide binding provides a bridging azide between Fe*_a_*_3_^3+^ and Cu_B_^2+^, in contrast to the above proposed structure. In general, an azide ion terminally bound to a ferric heme stabilizes the low-spin state, because the azide ion is a strong ligand to ferric heme. However, the bridging azide could promote the high-spin state in heme *a*_3_. The present X-ray structure is consistent with the resonance Raman results showing that the high-affinity azide binding provides the high-spin heme *a*_3_. Furthermore, the resonance Raman and X-ray structural results at a high-azide concentration suggest that the azide at Cu_B_^2+^ in the two azide-bound CcOs stabilizes the high-spin state in the terminally-bound azide derivative of the ferric heme *a*_3_.

The present results giving a binding mode ratio of 7 (bridging):3 (terminal) at 2 mm azide indicate that the *K_d_* value of the second step of the azide binding (formation of the two-azide–bound form from the one-azide–bound form) is 4.6 mm. This *K_d_* value obtained at pH 5.7 is consistent with the reported *K_d_* value of 42 mm (17) for the low-affinity site determined by absorption spectral analyses at pH 7.4 in solution, because the reported pH dependence of *K_d_* indicates that the *K_d_* value determined at pH 5.7 is about 1 order of magnitude less than the *K_d_* value determined at neutral pH (10). This consistency in the azide-binding affinity between solution and crystalline states suggests that the structure of the O_2_ reduction site in the physiological conditions (*i.e.* in solution state) is essentially preserved in the crystalline state, although the O_2_ transfer pathway to the O_2_ reduction site is significantly perturbed to decrease the azide-binding rate as described above.

### Structural coupling between the O_2_ reduction site and the H-pathway

The present X-ray structural analyses reveal that the second step of the azide binding to the resting oxidized CcO is accompanied by the azide-binding mode transition from the bridging to the terminal, which is undetectable by the absorption and Raman spectroscopic analyses. A simultaneous two azide binding directly to the resting oxidized state is unlikely because the spectral changes in absorbance for the azide binding to the low-affinity site (the second step) is consistent with an equilibrium, CcO–N_3_^−^ + N_3_^−^ = CcO–(N_3_^−^)_2_ (17). Thus, the bridging/terminal-binding mode transition induces the closed/open transition in the water channel without changing the oxidation and spin states of the O_2_ reduction site, mediated by the translational migration of heme *a*_3_ plane.

The conformation of the water channel is perturbed also by the coordination state change in Fe*_a_*_3_ in the fully reduced state of the O_2_ reduction site (*i.e.* release of CO from CcO-CO) to give an open conformation in the water channel, the structure of which is significantly different from the azide-induced open conformation (1, 5). The structural coupling is also mediated by the translational movement of heme *a*_3_ plane (4). These structural couplings between the O_2_ reduction site and the H-pathway support the critical involvement of the H-pathway as the proton pump system, driven by O_2_ reduction in the O_2_ reduction site.

### Possible inhibition mechanism by the azide binding to the low-affinity site

It has been proposed that protons for pumping are collected into a Mg^2+^-containing water cluster through the water channel of the H-pathway in the open state, which is attained when the O_2_ reduction site is in the fully reduced state (4). The water channel is closed upon O_2_ binding to Cu_B_ after the four protons were collected into the Mg^2+^-containing water cluster (21). The collected protons are transferred to the hydrogen-bond network of the H-pathway to be pumped to the P-side electrostatically driven by net positive charges created on heme *a* upon electron transfer to the O_2_ reduction site (4). The water channel closure determines the direction of the proton pump by blockage of the proton back-leak to the N-side. Thus, the opening of the water channel in the fully oxidized state upon azide binding to the low-affinity site would critically decrease the efficiency in the proton pump.

The channel opening is also likely to lower the level of protons to be pumped into the hydrogen-bond network under turnover conditions due to spontaneous proton leakage to the N-side. The proton level decrease is likely to decrease the electron affinity of heme *a* under the electrostatic influence from the hydrogen-bond network of the H-pathway. Then, the electron flow from Cu_A_ via heme *a* to the azide-bound Fe*_a_*_3_^3+^ would be decreased. It is well-known that azide does not bind to Fe*_a_*_3_^2+^ (12). Therefore, the suppression of the electron flow would increase the level of Fe*_a_*_3_^3+^–N_3_^−^ under turnover conditions to enhance the azide inhibition.

The electron affinity decrease in heme *a* is consistent with the reported redox potential decrease induced by azide at high concentration sufficient for saturating the low-affinity site (24, 25). Similarly, CO binding to Fe*_a_*_3_^2+^, accompanied by the channel closure, increases the potential (25). These results support the existence of the electrostatic influence of heme *a* to the hydrogen-bond network of the H-pathway. However, structural changes induced by the azide binding other than the channel opening is also likely to influence the redox potential of heme *a*. In fact, cyanide binding to Fe*_a_*_3_^3+^ decreases the redox potential without opening the channel (25), although the resolution (2.0 Å) of the crystal structure of cyanide-bound oxidized CcO (PDB code 3X2Q) is not sufficiently high for concluding that the water channel is completely closed. Moreover, the present X-ray structural analyses for the azide-bound CcO at 20 mm indicate that no significant structural change other than the channel opening is likely to influence the redox potential of heme *a*.

### Alternative proton-pumping mechanisms

The present results support the critical involvement of the H-pathway in the proton pump function of bovine CcO. However, it has been proposed based on the mutational and simulation analyses of bacterial CcOs as mentioned above that bacterial CcOs pump protons without using the H-pathway. In these mechanisms, both pumping and water-forming protons are transferred through a single proton-conducting pathway (either the D-pathway or the K-pathway) (1–3). In contrast, in the proton pump mechanism of bovine CcO, the proton pump pathway (H-pathway) is completely separated from the pathways for the water-forming protons. The difference should not be ignored, because the separation is prerequisite for the efficient energy transduction in CcO (26).

Recently, it has been proposed that the H-pathway does not function as the proton-pumping system by a simulation analysis using X-ray structural data of bovine CcO determined at 1.8 Å resolution (5), because access of the water molecules from the N-side to the water channel of the H-pathway is essentially impossible (27). His^413^ located near the entrance of the water channel, in the protonated state, stimulates penetration of water molecules from the N-side and forms a water wire through which protons are transferred to the hydrogen-bond network of the H-pathway. The residue in the deprotonated (or neutral) state strongly suppresses the water accumulation and disconnects the water wire. The latter simulation result, showing 5–6 water molecules in the channel region, is consistent with the X-ray structure at 1.8 Å resolution (5). However, 12 water molecules are resolved in the channel region in the X-ray structure at 1.6 Å resolution (4). These water molecules include all those proposed by stimulation assuming that His^413^ is protonated (27). These results indicate that the X-ray structure at 1.8 Å resolution (5) is not sufficiently high for identification of all the water molecules in the water channel detectable at 1.6 Å resolution (4). Reevaluations are desirable for the simulation analysis (27).

## Experimental procedures

### Preparation of azide-bound resting oxidized CcO crystals

The resting oxidized bovine heart CcO crystals were prepared as described before (15). Crystals were frozen at 90 K in 40 mm sodium phosphate buffer, pH 5.7, 0.2% decyl maltoside, 8% PEG 4000, 40% ethylene glycol, and 2–20 mm sodium azide. The final medium composition was attained by a 48 stepwise manual exchange from the initial medium composed of 40 mm sodium phosphate buffer, pH 6.5, 0.2% decyl maltoside, 1% PEG 4000, and 2% ethylene glycol in which the crystals are stable at 4 °C. The stepwise exchange of the medium was performed typically within 3 days (*i.e.* 20, 20, and 8 steps in the 1st, 2nd, and 3rd days, respectively). The azide concentration was gradually increased by adding increasing concentrations of azide into the media for the stepwise exchange up to the final concentration in the 1st day. The final azide concentration was retained over the following 2 days. Thus, the crystals were exposed for 2 days with azide at the final concentration. For attainment of a longer exposure time, the stepwise exchange was performed over a longer duration, but the azide concentration was increased to the final concentration within the 1st day. For example, a longer exposure was performed in the stepwise exchange in 5 days (12, 9, 9, 10, and 8 steps on each of the five consecutive days, respectively) concomitantly with a stepwise increase in azide concentration to the final concentration within the 1st day. In this paper, the azide exposure period (days) after attaining the final concentration is designated as the exposure time. Immediately after finishing the 48-step medium exchange simultaneously with azide exposure, the crystals were frozen at 90 K.

### X-ray diffraction experiments

Crystals for the X-ray diffraction experiments were prepared under four different conditions with respect to azide concentration and soaking period. All X-ray experiments were carried out at beamline BL44XU/SPring-8 equipped with an MAR300HE CCD detector. Crystals with sizes ∼700 × 700 × 200 μm were used in the diffraction experiments. In a representative series of diffraction measurements, the thin edge of a crystal was aligned parallel to the X-ray beam at a rotation angle of 0.0°. The wavelength was 0.9 Å, the photon number at the sample position was 4.0 × 10^11^ photons/s, and the crystal was shot with X-rays in a helium gas stream at 50 K and translated by 10 μm after each shot to reduce radiation damage. Other experimental conditions for low-resolution data collection were X-ray beam cross-section of 20 μm (vertical) × 20 μm (horizontal) at the crystal, a camera distance of 431 mm, exposure period of 1.0 s, and an oscillation angle of 1.0°. Conditions employed for high-resolution data were X-ray beam cross-section of 50 μm (vertical) × 30 μm (horizontal) at the crystal, a camera distance of 230 mm, exposure period of 3.0 s, and an oscillation angle of 0.5°. Crystalline absorption spectra were measured before and after the X-ray diffraction experiment with the spectrophotometer system as described before (28). The full occupancy of azide ions observed in the present X-ray structural results suggests that no significant structural damage in the azide-binding site is induced by X-ray irradiation, although the X-ray exposure in the present X-ray diffraction experimental conditions provides slight spectral changes assignable to heme *a* reduction. A total of 5–11 crystals were used for acquisition of the full data sets at 1.65 to 1.85 Å. Data processing and scaling were carried out using HKL2000 and SCALEPACK (29). A total of 678–851 images were successfully processed and scaled. The structure factor amplitude (|*F*|) was calculated using the CCP4 program TRUNCATE (30, 31). Other statistics obtained from the intensity data are provided in Table S1.

### X-ray structural determinations and refinements

Four types of crystals, exposed to 2 mm azide for 2 days (2mm_2days), to 20 mm azide for 2 days (20mm_2days), to 20 mm azide for 3 days (20mm_3days), and to 20 mm azide for 4 days (20mm_4days), prepared as described above (Table 5), have essentially identical cell dimensions within less than 0.2% deviation, indicating that all crystals are isomorphous with each other at the present resolution. The same procedures applied for the previous structural analyses of CcO crystals crystallized at pH 5.7 (4) were followed in the structural determinations. Initial phase angles of structure factors up to 4.0 Å resolution were calculated by the MR method (32) using the fully oxidized structure, previously determined at 1.5 Å resolution (PDB code 5B1A) (4). The phases were extended to each highest resolution by density modification (33) coupled with noncrystallographic symmetry (NCS) averaging (34, 35) using the CCP4 program DM (36). The resultant phase angles (α_MR/DM_) were used to calculate the electron-density map (MR/DM map) with Fourier coefficients |*F*| exp(*i*α_MR/DM_), where |*F*| is the observed structure factor amplitude.

Bulk solvent correction and anisotropic scaling of the observed and calculated structure factor amplitudes and TLS parameters were incorporated into the refinement calculation using REFMAC5 (37). The anisotropic *B*-factors for the iron, copper, and zinc atoms were imposed on the calculated structure factors. Each monomer of two NCS-related monomers was assigned to a single TLS group in the refinement. The crystal structure was refined under NCS restraints between two monomers. Ligands in the O_2_ reduction center, water molecules, ethylene glycol molecules, lipids, and detergents were located in an *F_o_* − *F_c_* map composed with the phases calculated with atomic parameters of protein atoms and cofactors.

Each *F_o_* − *F_c_* map was found to have significant electron density corresponding to azide ions. In the 20mm_4days map, two azide ions each terminally bound to Fe*_a_*_3_ and Cu_B_, respectively, were accommodated each with full occupancy of 1.0. In the 2mm_2days map, an azide group bridged between Fe*_a_*_3_ and Cu_B_. An additional *F_o_* − *F_c_* map was calculated by including the bridging azide group with occupancy of 1.0 to detect significant residual densities at the same sites as those of 20mm_4days. Because the bridging azide ions could not coexist with the terminally-bound azide ions, both types of azide ions were included in the refinement with their partial occupancies. The occupancies of azide ions were determined by inspecting the residual densities of the *F_o_* − *F_c_* map in each iteration of the refinement at a different occupancy of each 0.1 step. The structures of azide ions in the O_2_ reduction center of the other crystals were conducted by following these two cases.

When heme *a*_3_ structures were refined from the oxidized form determined at 1.5 Å resolution (PDB code 5B1A), they were converged to different locations from each other, and each of the average *B*-factor value of heme *a*_3_ was significantly higher than those of heme *a* of the same crystal. Further refinements for heme *a*_3_ were conducted with multiple structures. One structure was the same heme *a*_3_ structure as the resting oxidized form, and the other was a structure shifted further than that of the reduced form (PDB code 5B1B). Convergence of each structural refinement with respect to heme *a*_3_ was confirmed by inspecting *B*-factor values of heme *a*_3_ and the residual electron density at heme *a*_3_.

Significant residual density was found to exist at residues 380–383 of helix X in each *F_o_* − *F_c_* map, and *B*-factors of atoms of residues 380–383 were found to be higher than those of other atoms of helix X. The structures of residues 380–383 were refined by imposing multiple conformations. One conformation was the same structure as the oxidized form, and the other conformation was that of the reduced form. Convergency of each refinement with respect to helix X was confirmed as in the case of heme *a*_3_.

Although the *F_o_* − *F_c_* electron density of the water molecule, W1, hydrogen-bonded to Tyr^244^–OH was detected in 2mm_2days map, it was not detected in 20mm_4days map at the 3 σ level. The occupancies of the water molecule (W2) hydrogen-bonded to both Tyr^244^–OH and the OH group of the hydroxy farnesyl ethyl group of heme *a*_3_ and W1 were determined by inspecting each *B*-factor. To minimize the difference of the *B*-factors between W1 and Tyr^244^–OH, each refinement was performed with changing the occupancy of W1 in 5% step. In the case for W2, the differences between the *B*-factor values of W2 and the average of the *B*-factor values of Tyr^244^–OH and the hydroxy farnesyl ethyl OH group of heme *a*_3_ were examined for optimizing the occupancy value. Statistics of the refinements for these azide derivatives of CcO are summarized in Table S2.

## Author contributions

A. S., E. Y., K. M., T. T., and S. Y. conceptualization; A. S., K. H., H. T., N. Y., K. S.-I., E. Y., K. M., T. T., and S. Y. resources; A. S., K. H., H. T., N. Y., K. S.-I., E. Y., K. M., T. T., and S. Y. data curation; A. S., N. Y., E. Y., and T. T. software; A. S., K. H., H. T., N. Y., E. Y., T. T., and S. Y. formal analysis; A. S., K. S.-I., E. Y., K. M., T. T., and S. Y. supervision; A. S., T. T., and S. Y. funding acquisition; A. S., K. H., H. T., N. Y., E. Y., K. M., and T. T. validation; A. S., K. H., H. T., N. Y., K. S.-I., E. Y., K. M., T. T., and S. Y. investigation; A. S., K. H., H. T., N. Y., E. Y., K. M., and T. T. visualization; A. S., H. T., N. Y., K. S.-I., E. Y., T. T., and S. Y. methodology; A. S., T. T., and S. Y. writing-original draft; T. T. and S. Y. project administration.

## Supplementary Material

Supporting Information

## Footnotes

5 The abbreviations used are:

CcO: cytochrome *c* oxidase
heme *a* and heme *a*_3_: low and high-spin heme A molecules of CcO, respectively
Fe*_a_* and Fe*_a_*_3_: iron ions of heme *a* and heme *a*_3_, respectively
Cu_A_ and Cu_B_: low and high potential copper sites of CcO
H-pathway: the proton-pumping pathway of animal CcO
PDB: Protein Data Bank
MR: molecular replacement
DM: density modification
NCS: noncrystallographic symmetry.
