# Supplementary material for: X-ray structural analyses of azide-bound cytochrome c oxidases reveal that the H-pathway is critically important for the proton-pumping activity
Source: J Biol Chem. 2018 Aug 3;293(38):14868–79. doi: 10.1074/jbc.RA118.003123 (PMC6153300; doi:10.1074/jbc.RA118.003123)
Supplement: Supporting Information [file supp_293_38_14868__index.html]

X-ray structural analyses of azide–bound cytochrome c oxidases reveal that the H–pathway is critically important for the proton-pumping activity — Investigating proton pumping with cytochrome c oxidase–azide — X-ray structural analyses of azide-bound cytochrome c oxidases reveal that the H-pathway is critically important for the proton-pumping activity — Investigating proton pumping with cytochrome c oxidase–azide — Supporting Information 

# X-ray structural analyses of azide-bound cytochrome *c* oxidases reveal that the H-pathway is critically important for the proton-pumping activity

## Supporting Information

- X-ray structural analyses data statistics - X-ray diffraction data and refinement statistics
