# Supplementary material for: X-ray structural analyses of azide-bound cytochrome c oxidases reveal that the H-pathway is critically important for the proton-pumping activity
Source: J Biol Chem. 2018 Aug 3;293(38):14868–79. doi: 10.1074/jbc.RA118.003123 (PMC6153300; doi:10.1074/jbc.RA118.003123)
Supplement: Supporting Information [file supp_RA118.003123_136977_2_supp_175491_pcj0cg.pdf]

Supporting Information for JBC-2018-003123

Title: X-ray structural analyses of azide–bound cytochrome *c* oxidases reveal that the H–pathway is critically important for the proton-pumping activity

Authors: Atsuhiro Shimada[1¶], Keita Hatano[1], Hitomi Tadehara[1], Naomine Yano[1], Kyoko Shinzawa–Itoh[1], Eiki Yamashita[3], Kazumasa Muramoto[2‡], Tomitake Tsukihara[1,3,4‡], Shinya Yoshikawa[1‡]

Contents of this supporting information: Table S1 and Table S2

Table S1. X–ray diffraction data for fully oxidized azide–bound CcOs

Experimental conditions		
Beam line	BL44XU at the SPring–8	
Wavelength (Å)	0.9	
Detector	MAR300HE	
Temperature (K)	50	
Beam size		
High–resolution data	50 μm × 30 μm	
Low–resolution data	20 μm × 20 μm	
Crystal–Detector distance (mm)		
High–resolution data	230	
Low–resolution data	431	
Oscillation angle (˚)		
High–resolution data	0.5	
Low–resolution data	1.0	
Exposure period (sec.)		
High–resolution data	3.0	
Low–resolution data	1.0	
	2mM_2days	20mM_4days
Number of crystals		
High–resolution data	7	4
Low–resolution data	1	1
Diffraction data		
Space group	$P2_12_12_1$	$P2_12_12_1$
Cell dimensions of *a*, *b*, *c* (Å)	183.4, 206.7, 177.6	183.4, 206.3, 177.6
Resolution (Å) *	200.0–1.85 (1.87–1.85)	200.0–1.85 (1.87–1.85)
σ cutoff	–3.0	–3.0
Observed reflections	7,023,105	7,239,669
Independent reflections*	566,316 (14,080)	564,234 (13,836)
Averaged redundancy†*	12.4 (10.2)	12.8 (8.2)
$\langle I/\sigma(I)\rangle$ ‡*	35.3 (3.6)	42.5 (3.3)
Completeness*	99.9 (100.0)	99.7 (99.2)
R_{merge} #*	0.114 (1.128)	0.092 (1.082)

$R_{\mathrm{pim}}^{\$*}$	0.032 (0.364)	0.022 (0.391)
$CC_{1/2}^{*}$	(0.896)	(0.879)

[†] Redundancy is the number of observed reflections for each independent reflection.

[‡] $\langle I/\sigma(I)\rangle$ is the average of the intensity signal–to–noise ratio.

[#] $R_{\mathrm{merge}} = \Sigma_{hkl}\Sigma_i |I_i(hkl) - \langle I(hkl)\rangle| / \Sigma_{hkl}\Sigma_i I_i(hkl)$, where $I_i(hkl)$ is the intensity value of the ith measurement of *hkl*, and $\langle I(hkl)\rangle$ is the corresponding mean value of $I_i(hkl)$ for all *i* measurements. The summation is over reflections, with $I/\sigma(I)$ larger than −3.0.

$^{\$}$ $R_{\mathrm{pim}} = \Sigma_{hkl} (N-1)^{-1/2}\Sigma_i |I_i(hkl) - \langle I(hkl)\rangle| / \Sigma_{hkl}\Sigma_i I_i(hkl)$, where N is a multiplicity of each (*hkl*).

[*] Numbers in parentheses are given for the highest resolution shells.

Table S2. Statistics of refinements for fully oxidized azide–bound CcOs

	2mM_2days		20mM_4days	
Resolution(Å)*	40.0–1.90 (1.95–1.90)		40.0–1.85 (1.90–1.85)	
R††*	0.165 (0.219)		0.163 (0.252)	
R_{free}‡*	0.190 (0.249)		0.189 (0.260)	
r.m.s.d. §§ bonds (Å)*	0.0291		0.0289	
r.m.s.d. §§ angles (°)*	2.6		2.6	
Averaged B-factors (Å^2)				
	A	B	A	B
Protein atoms	37.7	43.4	39.8	45.7
Heavy metals	28.2	32.3	30.2	35.0
Lipids and detergents	73.7		77.3	
Waters	47.0		48.3	
All atoms	42.9		45.3	
Number of amino acid residues				
All residues	3,614		3,614	
Determined	3,558		3,558	
Multiple conformation	89		88	

†† R is the conventional crystallographic R factor, $R = \Sigma_{hkl} ||F_o| - |F_c|| / \Sigma_{hkl} |F_o|$, where F_o and F_c are the observed and calculated structure factors, respectively.

‡ R_{free} is the free R factor for the 5% of the reflections that were excluded from the refinement.

§§ Root–mean–square deviation.

*Numbers in parentheses are given for the highest resolution shells.

A and B indicate two independent enzyme molecules in an asymmetric unit.
